# Muscle quality index and hyperuricemia: adipose tissue as a mediator

**DOI:** 10.3389/fendo.2025.1562837

**Published:** 2025-05-27

**Authors:** Ya Shao, Yu Wang, Xuelian Jiang, Meiling Shao, Bin Liu, Longti Li, Huiqin Zhong

**Affiliations:** ^1^ Health Management Center, Wudangshan Branch, TaiHe Hospital, Hubei University of Medicine, Shiyan, Hubei, China; ^2^ Nursing Department, TaiHe Hospital, Hubei University of Medicine, Shiyan, Hubei, China; ^3^ Department of Pharmacy, TaiHe Hospital, Hubei University of Medicine, Shiyan, Hubei, China; ^4^ Innovation Centre of Nursing Research, TaiHe Hospital, Hubei University of Medicine, Shiyan, Hubei, China

**Keywords:** muscle quality index, serum uric acid, SUA, hyperuricemia, body fat percentage, visceral fat mass, adipose tissue, NHANES

## Abstract

**Objective:**

The relationship between the Muscle Quality Index (MQI) and hyperuricemia remains incompletely defined, and additional studies are necessary to elucidate the mediating role of adipose tissue in this association.

**Methods:**

This study utilized data from the 2011–2014 National Health and Nutrition Examination Survey (NHANES) and included 5,198 adults. MQI was calculated as the ratio of maximum handgrip strength to appendicular skeletal muscle mass (ASM), and adipose tissue parameters included body fat percentage (BF%) and visceral fat mass (VFM). To examine the link between MQI and hyperuricemia, multiple logistic regression models were employed, and generalized additive models were utilized to assess potential non-linear patterns. Mediation analysis was performed to assess the mediating effect of adipose tissue, and sensitivity analyses, which involved altering the thresholds for hyperuricemia and excluding individuals with major chronic conditions, were carried out to verify the consistency of the results.

**Results:**

When treated as a continuous variable, MQI demonstrated a strong inverse association with hyperuricemia. Each one-unit increment in MQI corresponded to a 50% decrease in the odds of hyperuricemia (OR: 0.50, 95% CI: 0.43–0.57). Quartile-based analysis revealed that individuals in the highest MQI quartile had a 68% lower odds of developing hyperuricemia compared to those in the lowest quartile (OR: 0.32, 95% CI: 0.25–0.41). Subgroup analyses confirmed this negative correlation across various strata, and sensitivity analyses showed consistent results. Mediation analysis indicated that BF% and VFM explained 49.32% and 53.62% of the association between MQI and hyperuricemia, respectively.

**Conclusion:**

This study reveals a significant negative correlation between MQI and hyperuricemia, mediated by adipose tissue. These findings suggest that improving muscle quality and managing adipose tissue could offer new strategies for mitigating hyperuricemia and promoting better public health outcomes.

## 1 Introduction

Hyperuricemia, marked by elevated serum uric acid (SUA), belongs to a class of metabolic conditions strongly linked to various cardiovascular and renal disorders (1–3). Over recent decades, lifestyle changes have contributed to a growing global burden of hyperuricemia, now affecting up to 20% of the U.S. population (4). The pathogenesis of hyperuricemia is complex, involving increased uric acid synthesis or impaired excretion, both of which are influenced by factors such as dietary patterns, obesity, insulin resistance, and lifestyle habits (5). Given its associations with various chronic diseases and adverse health outcomes, identifying actionable factors to prevent and manage hyperuricemia is an urgent priority for public health.

The Muscle Quality Index (MQI) is a composite measure used to assess muscle function by evaluating the strength produced relative to muscle mass (6). Compared to isolated assessments of muscle mass or strength, MQI provides a more comprehensive and sensitive approach to capturing muscle functionality (7, 8). However, existing research has revealed inconsistent associations between muscle functional parameters and SUA. Floriano et al. discovered a positive correlation between muscle mass and strength with SUA among kidney transplant patients, without significant functional capacity associations (9). Nahas et al. reported a positive correlation between muscle strength and SUA in a cohort of 2,361 elderly individuals (10). Conversely, Wen et al. observed an inverse relationship between MQI and SUA (11). Similarly, Liu et al.’s investigation of 4,236 Chinese individuals over 50 years demonstrated negative correlations between muscle mass, muscle strength, and SUA (12). These discrepancies likely stem from variations in population demographics, research design, and assessment methodologies, highlighting the potential limitations of evaluating muscle health through isolated muscle strength or mass parameters.

Muscle tissue serves as a critical metabolic nexus, actively participating in diverse metabolic processes, including glucose and fatty acid metabolism. Low muscle quality exhibits a profound association with insulin resistance, which is a well-established risk factor for multiple metabolic disorders (13). Insulin resistance is characteristically accompanied by chronic inflammation and oxidative stress, mechanisms that not only exacerbate insulin signal transduction impairments but potentially stimulate uric acid generation through increased reactive oxygen species production (14–16). These interconnected pathophysiological mechanisms may collectively contribute to elevated SUA generation, thereby further escalating metabolic dysregulation. Nonetheless, the link between MQI and hyperuricemia remains inadequately investigated.

Adipose tissue emerges as a complex endocrine organ, transcending its traditional role as a mere energy storage depot by secreting diverse bioactive substances that play a pivotal role in regulating systemic metabolism and inflammatory responses (17). Within the adipo-renal axis, adipokines such as leptin and adiponectin exert critical modulatory effects on renal function (18). The dynamic interconversion between brown adipose tissue and white adipose tissue potentially demonstrates a profound correlation with muscle function deterioration. As muscle function progressively declines, brown adipose tissue within the body may undergo conversion to white adipose tissue, a trans-differentiation phenomenon that potentially represents a key mechanism in metabolic disease progression (19). Excessive white adipose tissue can trigger multiple inflammatory responses, altering the body’s metabolic state and indirectly influencing muscle quality and uric acid metabolism (20). Irisin, a critical myokine secreted by skeletal muscle, plays a central role in energy metabolism regulation (21). By promoting the browning of white adipose tissue, it significantly increases energy expenditure and heat production, offering a novel potential pathway for combating obesity and metabolic-related disorders. Specifically, irisin activates p38 MAPK and ERK signaling pathways, promoting uncoupling protein 1 (UCP1) expression and fine-tuning energy metabolism (22). In patients with visceral fat accumulation, these metabolic pathways may undergo significant disruption, leading to metabolic dysfunction and aberrant energy homeostasis. Therefore, it is vital to explore the interaction of adipose tissue with muscle function and its role in metabolic dysregulation.

This study seeks to thoroughly examine the relationship between MQI and hyperuricemia, with a focus on the mediating role of adipose tissue, particularly body fat percentage (BF%) and visceral fat mass (VFM).

## 2 Materials and methods

### 2.1 Research design and population

This study utilized data from the 2011–2014 National Health and Nutrition Examination Survey (NHANES), a nationwide program aimed at assessing the health and nutritional status of the U.S. residents (23). To ensure the sample was representative, a multistage probability sampling technique was employed. Data collection involved administering questionnaires, conducting clinical assessments, and performing laboratory tests. The protocol received approval from the National Center for Health Statistics Ethics Review Board, and participants provided informed consent before involvement.

**Figure 1:**
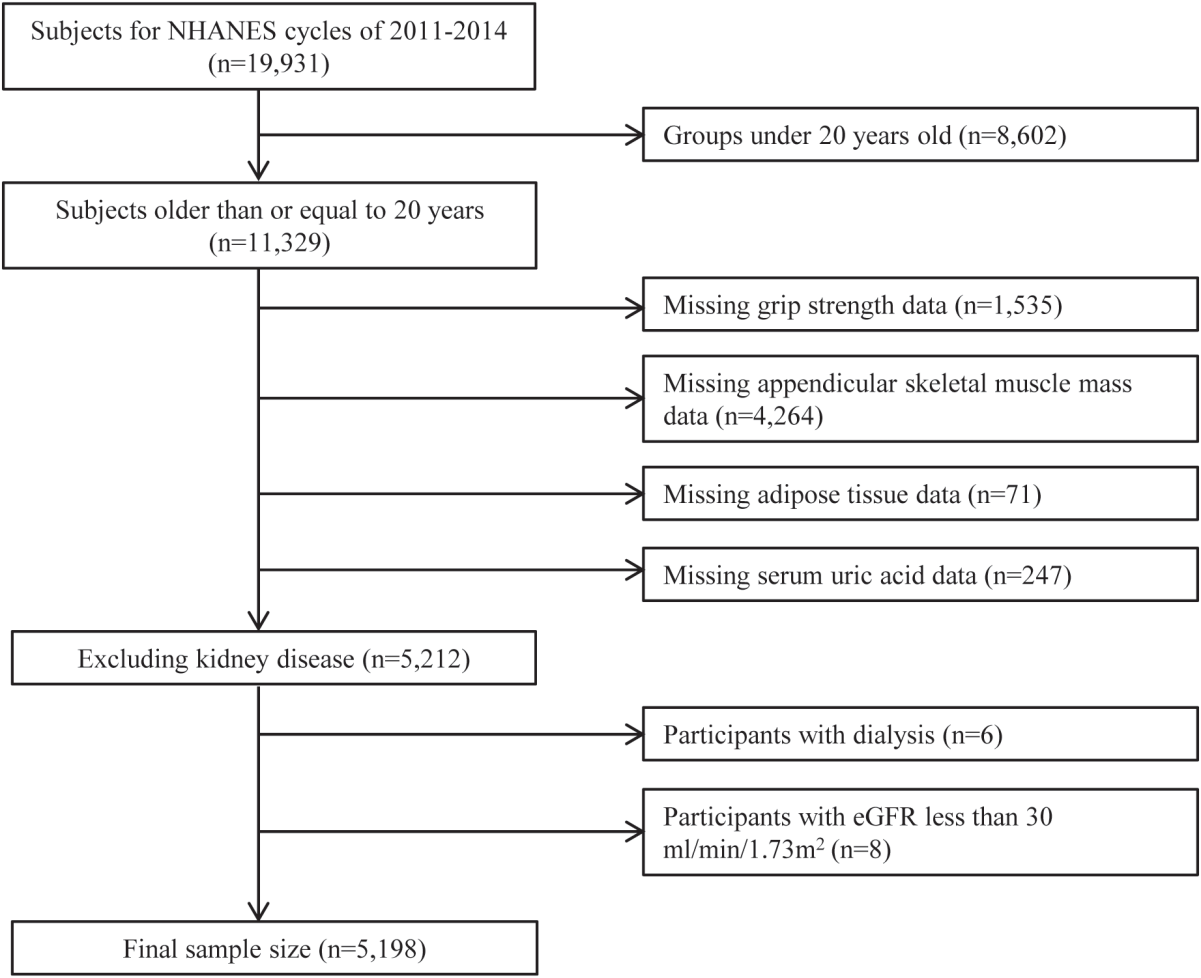
Participant selection flowchart for the study.

During the survey, 11,329 individuals aged 20 or older participated. We excluded individuals with missing key variables, including handgrip strength, appendicular skeletal muscle mass (ASM), and adipose tissue data (BF% and VFM), leaving 5,212 participants eligible for analysis. Furthermore, participants with severe kidney disease, including those who had undergone dialysis in the past year or severe renal dysfunction (estimated glomerular filtration rate [eGFR] <30 mL/min/1.73 m²), were also excluded. Ultimately, 5,198 participants remained (**Figure 1**).

### 2.2 Assessment of MQI and adipose tissue

MQI was determined by dividing the total maximum handgrip strength by ASM (24). Handgrip strength was assessed using a calibrated dynamometer. To ensure the reliability of the measurements, individuals undergoing recent surgical procedures on the hand or wrist within the previous three months were excluded. Trained examiners provided standardized instructions for the handgrip test, allowing each participant one practice attempt to ensure consistency and proper technique. The test was conducted three times per hand, with participants alternating hands and allowing one-minute rest periods between tests to minimize muscle fatigue. The maximum value recorded from each hand across the trials was used to calculate MQI.

Dual-energy X-ray absorptiometry was used to measure ASM and adipose tissue parameters, including BF% and VFM. This well-established technique offers precise assessments of skeletal muscle and fat. The system was calibrated daily, and scans were conducted by trained radiologic technicians. Expert radiologists reviewed the data to ensure quality control and consistency.

### 2.3 Assessment of hyperuricemia

SUA concentrations were determined from venous blood samples collected following a minimum fasting period of 9 hours. The collected blood was promptly frozen at -30°C and transported to a central laboratory for further analysis. Measurements of SUA were performed using a Beckman Coulter DxC800 automatic biochemical analyzer, utilizing a timed endpoint methodology (25). Hyperuricemia was defined as SUA >7.0 mg/dL in men and >6.0 mg/dL in women (26, 27), based on prior literature. For sensitivity analysis, an alternative definition using SUA ≥6.8 mg/dL was also employed (28).

### 2.4 Covariates

Based on existing literature (29, 30) and *a priori* knowledge, we comprehensively considered the potential influences of demographic characteristics, lifestyle factors, and clinical features on SUA to minimize potential confounding effects and interference. Demographic variables included sex, age, race, marital status, educational attainment, and poverty-to-income ratio (PIR), which were collected using standardized questionnaires. Definitions for physical activity (31), smoking (32), and alcohol consumption (33) were adapted from previously established literature. Liver function was evaluated by measuring alanine aminotransferase (ALT) levels, whereas kidney function was assessed using eGFR, calculated through the CKD-EPI formula based on serum creatinine (34). Chronic diseases considered included hypertension, diabetes, cardiovascular diseases (CVD), and cancer. These conditions were ascertained through self-reported medical histories, clinical documentation, or prescribed medication records. Hypertension and diabetes were additionally validated using measured blood pressure and glucose levels, respectively (35). CVD was defined as a reported diagnosis of heart failure, coronary artery disease, angina, myocardial infarction, or stroke (36).

### 2.5 Statistical analysis

Statistical analyses were performed using R software (V4.2.0) and EmpowerStats (V6.0). NHANES sampling design features, including sample weights, stratification, and clustering, were incorporated to ensure representativeness. To address incomplete covariate information—specifically involving missing data for education level (1 case), poverty-income ratio (PIR) (350 cases), physical activity (966 cases), smoking status (3 cases), drinking behavior (278 cases), and alanine aminotransferase (ALT) (1 case)—we employed a multiple imputation approach using chained equations (MICE). This method involved synthesizing five representative datasets for robust regression modeling. Baseline participant characteristics across MQI quartiles were summarized using descriptive statistics. Continuous variables are reported as means with standard errors (SE) and compared using analysis of variance, while categorical variables are expressed as frequencies and percentages, with group differences assessed via chi-square tests.

Logistic regression models were utilized to examine the relationship between MQI and hyperuricemia, with MQI assessed both as a continuous variable and by quartiles. Four models were developed: Model 1 was unadjusted; Model 2 included adjustments for sex, age, and race; Model 3 incorporated additional adjustments for marital status, educational attainment, PIR, physical activity, alcohol intake, smoking habits, ALT, and eGFR, while Model 4 included full adjustments additionally for hypertension, diabetes, CVD, and cancer. To investigate potential non-linear associations, a generalized additive mixed model was employed (37).

Subgroup analyses were performed to assess potential effect modifications across different population characteristics, incorporating interaction terms for evaluation. Sensitivity analyses were performed using alternative criteria for defining hyperuricemia (SUA ≥6.8 mg/dL), treating SUA as a continuous measure, excluding participants with gout or other chronic diseases (hypertension, diabetes, CVD, and cancer), and excluding all cases with missing covariates. Linear regression examined the association between MQI and adipose tissue parameters, while logistic regression assessed the link between adipose tissue and hyperuricemia.

Mediation analysis was conducted using the R mediation package following the approach proposed by Imai et al. (38). Regression models estimated relationships among the independent variable, mediator, and dependent variable using a generalized linear model with a probit link function. Indirect, direct, and total effects were quantified, with significance assessed through non-parametric bootstrapping (1,000 resamples) to evaluate the mediator’s role in the relationship. To address potential unmeasured confounding, we examined the correlation of residuals between the mediator and outcome models to assess the assumption of no unmeasured confounding. The extremely low residual correlation (0.004) indicates that the key assumption of no significant unmeasured confounding was satisfied, supporting the validity of the mediation analysis.

## 3 Results

### 3.1 Characteristics of the participants according to quartiles of MQI

In total, 5,198 individuals were analyzed, with 48.48% identified as female and an average age of 39.37 years. Among them, 830 individuals (15.57%) were classified as having hyperuricemia. **Table 1** summarizes the characteristics of individuals categorized by MQI quartiles. Those in the upper MQI quartiles were more likely to be male, identified as Non-Hispanic White, have higher educational levels, and participate in vigorous physical activities. Additionally, differences in smoking and alcohol consumption were observed between the groups. Participants with higher MQI also had significantly lower rates of hypertension, diabetes, and CVD. Higher MQI was also associated with younger age, greater PIR and eGFR, as well as reduced BF%, VFM, and SUA. No notable differences in ALT levels were detected across MQI quartiles.

**Table 1:** Weighted characteristics of participants across quartiles of muscle quality index (n = 5,198).

Characteristic	Muscle quality index				*P*-value
	Q1 (<2.96)	Q2 (2.96-3.38)	Q3 (3.39-3.81)	Q4 (≥3.82)	
Sex, n (%)					<0.001
Male	543 (41.61)	656 (50.65)	710 (53.67)	774 (58.44)	
Female	774 58.39)	661 (49.35)	607 (46.33)	544 (41.56)	
Age, years, mean (SE)	41.04 (0.66)	40.12 (0.54)	38.83 (0.55)	37.62 (0.43)	0.002
Race, n (%)					<0.001
Mexican American	164 (9.73)	177 (9.89)	159 (9.07)	179 (10.96)	
Other Hispanic	103 (6.05)	143 (7.73)	118 (6.11)	104 (5.90)	
Non-Hispanic White	463 (59.24)	523 (64.73)	559 (67.64)	543 (64.77)	
Non-Hispanic Black	447 (18.67)	280 (10.78)	222 (8.16)	173 (6.92)	
Other Race	140 (6.30)	194 (6.87)	259 (9.02)	319 (11.45)	
Education, n (%)					<0.001
Under high school	232 (14.03)	223 (12.70)	204 (12.03)	261 (16.29)	
High school or equivalent	313 (23.62)	261 (18.65)	254 (18.81)	290 (22.70)	
Above high school	772 (62.35)	833 (68.65)	859 (69.16)	767 (61.01)	
MET sore, n (%)					0.002
<600	197 (22.48)	167 (15.36)	155 (15.89)	185 (17.98)	
600∼3000	407 (40.25)	472 (43.43)	465 (43.07)	408 (36.66)	
≥3000	400 (37.27)	478 (41.20)	453 (41.04)	497 (45.36)	
Smoking, n (%)					0.002
Never	806 (59.83)	802 (58.92)	800 (59.05)	739 (55.11)	
Former	211 (17.33)	231 (21.82)	230 (20.10)	207 (16.76)	
Current	300 (22.84)	284 (19.26 )	287 (20.85)	372 (28.13)	
Alcohol, n (%)					0.004
Yes	963 (78.68)	1024 (84.52)	1046 (83.75)	1006 (83.42)	
No	354 (21.32)	293 (15.48)	271 (16.25)	312 (16.58)	
Gout, n (%)					0.147
Yes	35 2.77)	31 (2.58)	31 (2.47)	15 (1.00)	
No	1282 (97.23)	1286 (97.42)	1286 (97.53)	1303 (99.00)	
Hypertension, n (%)					<0.001
Yes	492 (34.57)	369 (28.04)	292 (21.33)	234 (19.09)	
No	825 (65.43)	948 (71.96)	1025 (78.67)	1084 (80.91)	
Diabetes, n (%)					<0.001
Yes	241 (15.62)	135 (8.72)	86 (4.97)	46 (2.34)	
No	1076 (84.38)	1182 (91.28)	1231 (95.03)	1272 (97.66)	
CVD, n (%)					0.003
Yes	77 (5.02)	46 (3.00)	38 (2.57)	28 (2.04)	
No	1240 (94.98)	1271 (97.00)	1279 (97.43)	1290 (97.96)	
Cancer, n (%)					0.745
Yes	51 (4.77)	55 (5.84)	47 (4.50)	44 (5.55)	
No	1266 (95.23)	1262 94.16)	1270 (95.50)	1274 (94.45)	
PIR, mean (SE)	2.76 (0.08)	2.97 (0.10)	3.04 (0.10)	2.93 (0.11)	0.029
ALT, U/L, mean (SE)	27.67 (0.68)	26.64 (0.63)	25.72 (0.61)	25.26 (0.73)	0.162
eGFR, ml/min/1.73m^2^, mean (SE)	100.86 (0.55)	102.17 (0.57)	102.28 (0.67)	103.71 (0.63)	0.023
BF, %, mean (SE)	37.44 (0.39)	33.47 (0.26)	31.57 (0.35)	29.42 (0.31)	<0.001
VFM, 100g, mean (SE)	6.52 (0.15)	5.25 (0.12)	4.54 (0.09)	3.78 (0.08)	<0.001
SUA, mg/dL, mean (SE)	5.5 (0.06)	5.42 (0.04)	5.31 (0.05)	5.16 (0.05)	<0.001

CVD, cardiovascular disease; PIR, poverty income ratio; ALT, alanine aminotransferase; eGFR, estimated glomerular filtration rate; BF, fat percentage; VFM, visceral fat mass; SUA, serum uric acid.

### 3.2 Association of MQI with hyperuricemia

**Figure 2:**
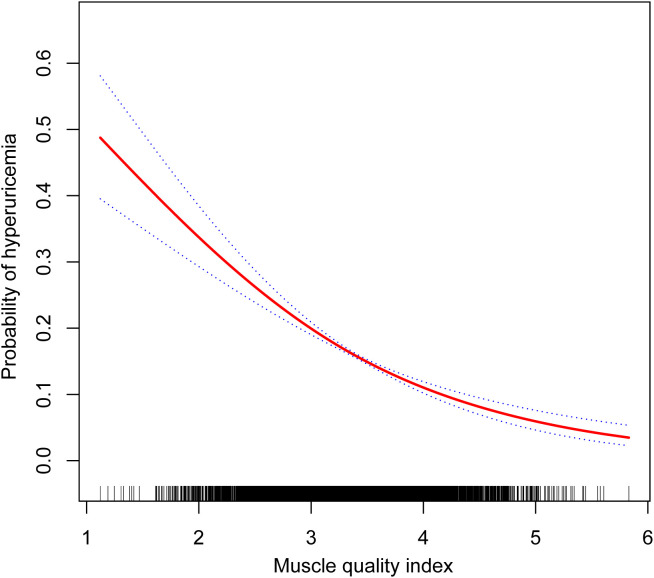
Dose-response relationship between muscle quality index and hyperuricemia.

The evaluation of MQI as a continuous variable consistently demonstrated a notable inverse relationship with hyperuricemia across all models. In Model 4, every one-unit increment in MQI was linked to a 50% decrease in the likelihood of developing hyperuricemia (OR: 0.50, 95% CI: 0.43–0.57). When MQI was analyzed by quartiles, the inverse association with hyperuricemia was further supported. As MQI advanced from Q1 to Q4, the probability of hyperuricemia decreased significantly (P for trend <0.001). Individuals within the highest quartile (Q4) exhibited a 68% lower likelihood of hyperuricemia compared to Q1 (OR: 0.32, 95% CI: 0.25–0.41) (**Table 2**). Furthermore, the generalized additive mixed-effects model illustrated a linear inverse relationship between MQI and hyperuricemia (**Figure 2**).

**Table 2:** Association between muscle quality index and hyperuricemia (n = 5,198).

Variables	Model 1	Model 2	Model 3	Model 4
	OR (95%CI)	OR(95%CI)	OR (95%CI)	OR (95%CI)
MQI as continuous	0.54 (0.48, 0.61)	0.47 (0.41, 0.53)	0.47 (0.41, 0.54)	0.50 (0.43, 0.57)
MQI as quartile				
Q1	1.00 (ref)	1.00 (ref)	1.00 (ref)	1.00 (ref)
Q2	0.65 (0.54, 0.79)	0.59 (0.49, 0.73)	0.60 (0.49, 0.74)	0.62 (0.50, 0.76)
Q3	0.53 (0.43, 0.65)	0.45 (0.37, 0.56)	0.46 (0.37, 0.57)	0.48 (0.39, 0.60)
Q4	0.36 (0.29, 0.45)	0.29 (0.23, 0.37)	0.30 (0.24, 0.38)	0.32 (0.25, 0.41)
* P* for trend	<0.001	<0.001	<0.001	<0.001

OR, Odds Ratio; CI, confidence interval; MQI, muscle quality index.

### 3.3 Subgroup and sensitivity analysis

**Figure 3:**
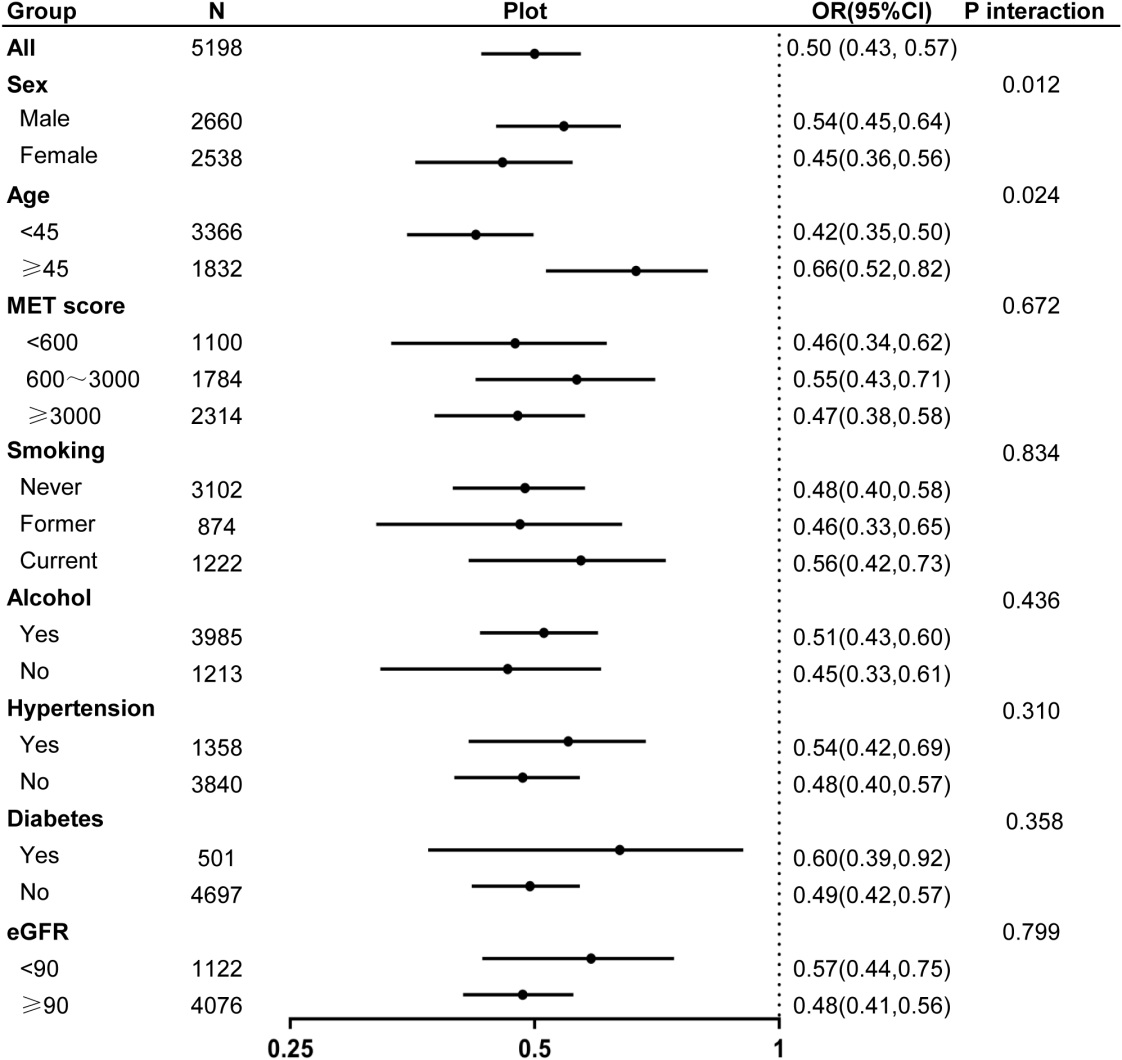
Forest plot of odds ratios for hyperuricemia associated with muscle quality index across different subgroups.

Subgroup analyses based on demographic and clinical characteristics, including sex, age, activity levels, smoking habits, alcohol consumption, eGFR, and hypertension or diabetes, consistently demonstrated an inverse association between MQI and hyperuricemia (**Figure 3**). Notably, an interaction effect was observed in the subgroup analysis by sex and age, with a stronger association found in females and individuals younger than 45 years (P for interaction < 0.05).

Sensitivity analyses using different SUA cut-off values, analyzing SUA as a continuous variable, and repeating the regression analysis excluding participants with gout or chronic diseases all supported the negative association between MQI and hyperuricemia. Furthermore, analyses performed without MICE also showed consistent results (**Supplementary Tables S1**-**S5**).

### 3.4 Associations of adipose tissue with MQI and hyperuricemia

Regression analysis identified significant inverse relationships between MQI and both BF% and VFM. For BF%, analyzing MQI as a continuous variable revealed that per-unit of MQI corresponded to a decrease in BF% by β = -3.64 (95% CI: -3.89, -3.40). When MQI was divided into quartiles, individuals in the top quartile (Q4) demonstrated a greater reduction in BF% compared to those in Q1, with an effect size of β = -5.86 (95% CI: -6.28, -5.44). Similarly, for VFM, each additional unit of MQI corresponded to a decrease in VFM by β = -1.47 (95% CI: -1.56, -1.37). When stratified by quartiles, the reduction in VFM for Q4 relative to Q1 was β = -2.37 (95% CI: -2.54, -2.21) (**Supplementary Table S6**).

Further analysis identified a strong positive association between both BF% and VFM and the likelihood of hyperuricemia. For BF%, every unit increase in BF% led to a 3% rise in the probability of hyperuricemia (OR = 1.03, 95% CI: 1.02, 1.04). When BF% was stratified into quartiles, individuals in the top quartile (Q4) had an 89% higher probability of hyperuricemia compared to those in the lowest quartile (Q1) (OR = 1.89, 95% CI: 1.49, 2.41). As for VFM, each unit increase in VFM was associated with a higher probability of hyperuricemia (OR = 1.33, 95% CI: 1.28, 1.38). Quartile-based analysis revealed that individuals in Q4 exhibited a nearly tenfold higher risk of hyperuricemia than those in Q1 (OR = 9.91, 95% CI: 7.21, 13.62) (**Supplementary Table S7**).

### 3.5 Mediating role of adipose tissue

**Figure 4:**
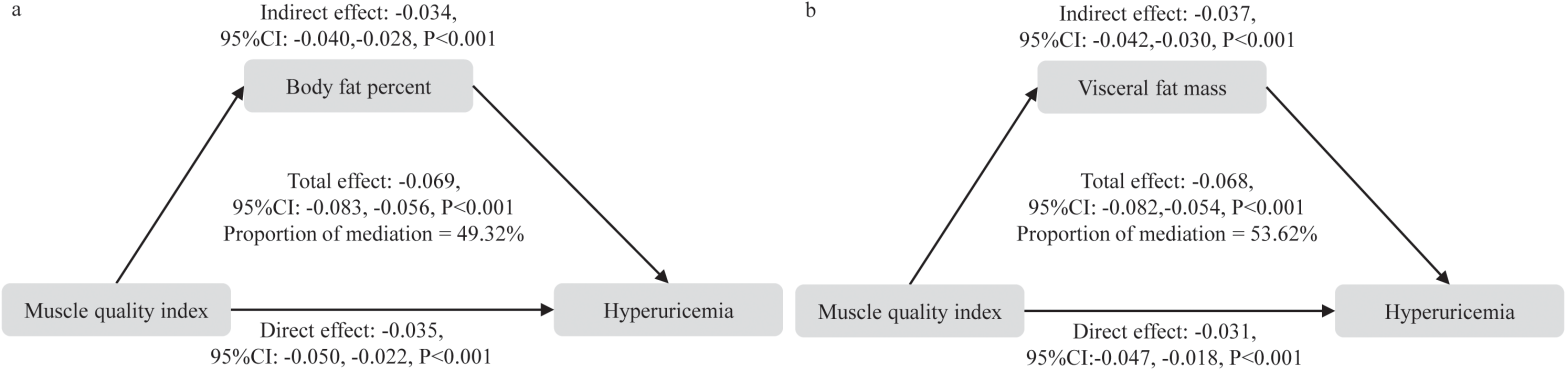
Mediation analysis of body fat percent and visceral fat mass in the relationship between muscle quality index and hyperuricemia. **(a)** body fat percentage, **(b)** visceral fat mass.

We further analyzed the mediating role of adipose tissue (including BF% and VFM) in the association between MQI and hyperuricemia. When BF% was used as the mediator, BF% explained 49.32% of the association between MQI and hyperuricemia. When VFM was used as the mediator, a similar mediation effect was observed, with an effect size of 53.62% (**Figure 4**).

## 4 Discussion

This study, utilizing NHANES data, systematically explored the link between MQI and hyperuricemia and identified a robust inverse association between the two. This association remained consistent despite controlling for various potential confounders, including age, sex, lifestyle factors, and chronic health conditions, indicating the important contribution of muscle function to uric acid regulation. Furthermore, the study underscores the intermediary effect of adipose tissue in this association, offering new insights into the intricate relationships between muscle, fat, and uric acid metabolism.

MQI, as a comprehensive indicator reflecting the strength produced per unit of muscle mass, offers unique advantages in assessing muscle functionality and metabolic capacity. It incorporates the rate of muscle shortening and reflects the quality of neuromuscular control (39). Muscle tissue plays a critical role not only in motor function but also in metabolic regulation, influencing a wide array of physiological and psychological health outcomes, including respiratory diseases (40), heart failure (41), cancer prognosis (42), and periodontal disease (43). Several studies have shown that lower MQI values correlate with depression (44, 45) and sleep disorders (46). Nevertheless, the connection between MQI and hyperuricemia has been relatively understudied in existing literature.

Earlier research has focused primarily on muscle mass or muscle strength in isolation with respect to SUA. Several studies have reported an inverse association, where higher SUA levels correlate with lower muscle mass or strength. For instance, Xu et al. observed that older adults with hyperuricemia are more prone to experiencing a decline in muscle quality and strength (47). Similarly, Liu et al. identified a negative association between elevated SUA levels and sarcopenia in a cohort of Chinese individuals aged over 50 years (12). On the other hand, other studies have proposed that increased SUA levels could offer advantages for maintaining muscle function. One study demonstrated that higher SUA in middle-aged and elderly Chinese individuals is linked with larger muscle mass (48). Furthermore, studies on elderly populations in Korea and the United States has demonstrated a positive relationship between SUA and grip strength (49, 50). Huang et al. also observed that higher SUA levels were associated with a reduced risk of muscle strength decline in elderly women (51).

The inconsistencies in these findings may stem from focusing on muscle mass or individual muscle strength measures, rather than taking a comprehensive approach to assessing muscle function. Traditional muscle mass indicators, such as ASM, often focus on muscle volume and may fail to capture the decline in muscle function, such as functional muscle loss in sarcopenia. In contrast, MQI, by combining muscle strength and mass, offers a more comprehensive metric and can better reflect muscle functionality and overall health changes (52). In this study, we observed a significant inverse relationship between MQI and hyperuricemia. Each additional unit increase in MQI was linked to a 50% reduction in the likelihood of hyperuricemia, a finding that remained consistent across subgroup and sensitivity analyses. Notably, this study identified a linear negative correlation between MQI and hyperuricemia, suggesting that higher MQI levels may contribute to improving uric acid metabolism.

The potential mechanisms underlying the association between MQI and hyperuricemia include muscle being a metabolically active tissue that participates in several metabolic processes. Low muscle quality may induce insulin resistance (53), which suppresses uric acid excretion in the kidneys, leading to hyperuricemia (54). Low muscle quality may reflect mitochondrial dysfunction and reduced metabolic efficiency, leading to the accumulation of purine metabolites and increased uric acid production (55). Animal experiments have also shown that the degradation of purines in skeletal muscle may contribute to elevated uric acid levels (56).

Recent studies by Wen et al., observed an inverse association between SUA and MQI (11). Our study corroborates this finding but also provides additional insights, especially regarding the mediating effect of adipose tissue in the connection between MQI and hyperuricemia. BF% and VFM accounted for nearly half of this association.

Adipose tissue serves as a crucial endocrine organ, releasing various adipokines (including leptin, adiponectin, TNF-α, and IL-6), which are pivotal in regulating metabolism, immune responses, and inflammation (57, 58). The accumulation of adipose tissue significantly increases the levels of pro-inflammatory cytokines, activating systemic inflammation and affecting uric acid production. Additionally, visceral fat accumulation is closely linked to kidney fat infiltration, representing a structural alteration that could influence renal uric acid excretion (59). Moreover, adipose tissue is not only a significant risk factor for disorders in uric acid metabolism but also exacerbates this risk through its interaction with muscle function. Excessive adiposity generates a pro-inflammatory environment, leading to widespread metabolic dysfunction, hindering muscle regeneration, and ultimately causing muscle quality and function loss, which aggravates metabolic health (60). This study underscores that individuals with low MQI are typically linked to higher BF% and VFM levels, which further supports the notion that low MQI may indirectly increase the likelihood of hyperuricemia through adipose tissue accumulation.

This study has important public health and clinical implications. By revealing the negative correlation between MQI and hyperuricemia, it suggests that improving muscle function may become an effective strategy for reducing the likelihood of hyperuricemia. Moreover, this research is the first to systematically analyze the mediating effect of adipose tissue in the connection between MQI and hyperuricemia, offering a potential theoretical foundation for clinical approaches that integrate fat management with muscle health.

The strengths of this study stem from its utilization of the highly representative NHANES database and the incorporation of subgroup and sensitivity analyses, particularly concerning varying hyperuricemia thresholds, which enhances the reliability of the findings. Nonetheless, several limitations should be acknowledged. First, the inherent temporal ambiguity in cross-sectional mediation analysis fundamentally constrains causal inference, rendering it impossible to definitively establish the chronological sequence of metabolic changes. Despite adjusting for numerous potential confounders, the cross-sectional design of this study precludes any definitive conclusions regarding causal relationships between MQI and hyperuricemia. While our analysis suggests potential mediating pathways through adipose tissue, the single time-point measurement may oversimplify complex metabolic interactions and fail to capture dynamic physiological processes. Longitudinal studies are essential to validate these observations further. Moreover, the measurement of body composition limited to participants younger than 60 in the NHANES survey significantly restricts the generalizability of our findings across diverse age demographics. Second, while the mediating role of adipose tissue was established in this study, its precise mechanisms still require deeper investigation through more comprehensive experimental approaches. Future research should prioritize prospective cohort studies, controlled experimental designs, and advanced molecular tracking techniques to elucidate the nuanced relationships between MQI, adipose tissue metabolism, and hyperuricemia.

## 5 Conclusions

Overall, this study reveals a strong inverse relationship between MQI and hyperuricemia, with adipose tissue playing a key mediating role in this connection. These results offer novel perspectives on the intricate relationship between muscle function, fat accumulation, and uric acid metabolism, highlighting the reciprocal effects of muscle health and fat storage, and suggesting potential avenues for managing hyperuricemia.

## Data Availability

Publicly available datasets were analyzed in this study. This data can be found here: https://wwwn.cdc.gov/nchs/nhanes/default.aspx.
