# Supplementary material for: Muscle quality index and hyperuricemia: adipose tissue as a mediator
Source: Front Endocrinol (Lausanne). 2025 May 27;16:1562837. doi: 10.3389/fendo.2025.1562837 (PMC12148888; doi:10.3389/fendo.2025.1562837)
Supplement: Supplementary file 1 [file Table1.docx]

Supplementary Material

# Supplementary Tables

**Supplementary Table S1.** Sensitivity analysis of the relationship between muscle quality index and hyperuricemia (SUA ≥6.8 mmol/L) (n = 5,198).

| **Variables** | **Model 1** |  | **Model 2** |  | **Model 3** |  | **Model 4** |
| --- | --- | --- | --- | --- | --- | --- | --- |
|  | **OR (95%CI)** |  | **OR(95%CI)** |  | **OR (95%CI)** |  | **OR (95%CI)** |
| **MQI as continuous** | 0.70 (0.62, 0.79) |  | 0.51 (0.44, 0.59) |  | 0.51 (0.44, 0.59) |  | 0.53 (0.46, 0.62) |
| **MQI as quartile** |  |  |  |  |  |  |  |
| Q1 | 1.00 (ref) |  | 1.00 (ref) |  | 1.00 (ref) |  | 1.00 (ref) |
| Q2 | 0.82 (0.66, 1.00) |  | 0.65 (0.52, 0.81) |  | 0.65 (0.52, 0.82) |  | 0.68 (0.54, 0.85) |
| Q3 | 0.76 (0.62, 0.94) |  | 0.54 (0.43, 0.68) |  | 0.53 (0.42, 0.67) |  | 0.56 (0.44, 0.71) |
| Q4 | 0.56 (0.44, 0.69) |  | 0.35 (0.28, 0.45) |  | 0.35 (0.28, 0.46) |  | 0.38 (0.29, 0.49) |
| *P* for trend | <0.001 |  | <0.001 |  | <0.001 |  | <0.001 |

Abbreviations: OR Odds Ratio, CI confidence interval, MQI muscle quality index.

**Supplementary Table S2.** Sensitivity analysis of the relationship between muscle quality index and serum uric acid (continuous variable) (n = 5,198).

| **Variables** | **Model 1** |  | **Model 2** |  | **Model 3** |  | **Model 4** |
| --- | --- | --- | --- | --- | --- | --- | --- |
|  | **β (95%CI)** |  | **β (95%CI)** |  | **β (95%CI)** |  | **β (95%CI)** |
| **MQI as continuous** | -0.24 (-0.29, -0.18) |  | -0.40 (-0.45, -0.35) |  | -0.36 (-0.41, -0.31) |  | -0.34 (-0.39, -0.29) |
| **MQI as quartile** |  |  |  |  |  |  |  |
| Q1 | 1.00 (ref) |  | 1.00 (ref) |  | 1.00 (ref) |  | 1.00 (ref) |
| Q2 | -0.12 (-0.22, -0.01) |  | -0.24 (-0.33, -0.15) |  | -0.21 (-0.30, -0.13) |  | -0.20 (-0.29, -0.12) |
| Q3 | -0.24 (-0.34, -0.14) |  | -0.43 (-0.52, -0.34) |  | -0.41 (-0.49, -0.32) |  | -0.38 (-0.47, -0.29) |
| Q4 | -0.38 (-0.48, -0.27) |  | -0.63 (-0.72, -0.54) |  | -0.57 (-0.66, -0.49) |  | -0.54 (-0.63, -0.45) |
| *P* for trend | <0.001 |  | <0.001 |  | <0.001 |  | <0.001 |

Abbreviations: CI confidence interval, MQI muscle quality index.

**Supplementary Table S3.** Sensitivity analysis of the relationship between muscle quality index and hyperuricemia for missing covariates without multiple imputation by chained equations (n = 3,777).

| **Variables** | **Model 1** |  | **Model 2** |  | **Model 3** |  | **Model 4** |
| --- | --- | --- | --- | --- | --- | --- | --- |
|  | **OR (95%CI)** |  | **OR(95%CI)** |  | **OR (95%CI)** |  | **OR (95%CI)** |
| **MQI as continuous** | 0.54 (0.46, 0.62) |  | 0.45 (0.39, 0.53) |  | 0.46 (0.39, 0.55) |  | 0.49 (0.41, 0.58) |
| **MQI as quartile** |  |  |  |  |  |  |  |
| Q1 | 1.00 (ref) |  | 1.00 (ref) |  | 1.00 (ref) |  | 1.00 (ref) |
| Q2 | 0.64 (0.51, 0.81) |  | 0.59 (0.47, 0.74) |  | 0.60 (0.47, 0.77) |  | 0.62 (0.49, 0.79) |
| Q3 | 0.52 (0.41, 0.66) |  | 0.43 (0.34, 0.56) |  | 0.44 (0.34, 0.57) |  | 0.47 (0.36, 0.61) |
| Q4 | 0.37 (0.28, 0.48) |  | 0.29 (0.22, 0.38) |  | 0.30 (0.22, 0.39) |  | 0.32 (0.24, 0.42) |
| *P* for trend | <0.001 |  | <0.001 |  | <0.001 |  | <0.001 |

Abbreviations: OR Odds Ratio, CI confidence interval, MQI muscle quality index.

**Supplementary Table S4.** Sensitivity analysis of the relationship between muscle quality index and hyperuricemia, excluding gout patients (n = 5,089).

| **Variables** | **Model 1** |  | **Model 2** |  | **Model 3** |  | **Model 4** |
| --- | --- | --- | --- | --- | --- | --- | --- |
|  | **OR (95%CI)** |  | **OR(95%CI)** |  | **OR (95%CI)** |  | **OR (95%CI)** |
| **MQI as continuous** | 0.54 (0.48, 0.61) |  | 0.47 (0.41, 0.54) |  | 0.47 (0.41, 0.54) |  | 0.50 (0.43, 0.57) |
| **MQI as quartile** |  |  |  |  |  |  |  |
| Q1 | 1.00 (ref) |  | 1.00 (ref) |  | 1.00 (ref) |  | 1.00 (ref) |
| Q2 | 0.63 (0.51, 0.76) |  | 0.57 (0.46, 0.70) |  | 0.57 (0.47, 0.71) |  | 0.59 (0.48, 0.73) |
| Q3 | 0.52 (0.42, 0.64) |  | 0.44 (0.36, 0.55) |  | 0.45 (0.36, 0.56) |  | 0.47 (0.37, 0.59) |
| Q4 | 0.36 (0.29, 0.46) |  | 0.30 (0.23, 0.37) |  | 0.30 (0.24, 0.39) |  | 0.32 (0.25, 0.41) |
| *P* for trend | <0.001 |  | <0.001 |  | <0.001 |  | <0.001 |

Abbreviations: OR Odds Ratio, CI confidence interval, MQI muscle quality index.

**Supplementary Table S5.** Sensitivity analysis of the relationship between muscle quality index and hyperuricemia, excluding participants with chronic diseases (n = 3,040).

| **Variables** | **Model 1** |  | **Model 2** |  | **Model 3** |
| --- | --- | --- | --- | --- | --- |
|  | **OR (95%CI)** |  | **OR(95%CI)** |  | **OR (95%CI)** |
| **MQI as continuous** | 0.58 (0.49, 0.69) |  | 0.48 (0.40, 0.57) |  | 0.50 (0.41, 0.60) |
| **MQI as quartile** |  |  |  |  |  |
| Q1 | 1.00 (ref) |  | 1.00 (ref) |  | 1.00 (ref) |
| Q2 | 0.63 (0.48, 0.83) |  | 0.54 (0.41, 0.72) |  | 0.56 (0.42, 0.76) |
| Q3 | 0.53 (0.40, 0.70) |  | 0.42 (0.31, 0.56) |  | 0.43 (0.32, 0.58) |
| Q4 | 0.39 (0.29, 0.52) |  | 0.28 (0.21, 0.38) |  | 0.30 (0.22, 0.42) |
| *P* for trend | <0.001 |  | <0.001 |  | <0.001 |

Abbreviations: OR Odds Ratio, CI confidence interval, MQI muscle quality index.

**Supplementary Table S6.** Association of muscle quality index with body fat percentage and visceral fat mass (n = 5,198).

| **Variables** | **Model 1** |  | **Model 2** |  | **Model 3** |  | **Model 4** |
| --- | --- | --- | --- | --- | --- | --- | --- |
|  | **β (95%CI)** |  | **β (95%CI)** |  | **β (95%CI)** |  | **β (95%CI)** |
| **For BF%** |  |  |  |  |  |  |  |
| **MQI as continuous** | -5.17 (-5.51, -4.83) |  | -3.90 (-4.15, -3.66) |  | -3.84 (-4.07, -3.60) |  | -3.64 (-3.89, -3.40) |
| **MQI as quartile** |  |  |  |  |  |  |  |
| Q1 | 1.00 (ref) |  | 1.00 (ref) |  | 1.00 (ref) |  | 1.00 (ref) |
| Q2 | -3.94 (-4.55, -3.33) |  | -3.02 (-3.43, -2.60) |  | -2.98 (-3.39, -2.57) |  | -2.83 (-3.24, -2.42) |
| Q3 | -6.23 (-6.83, -5.62) |  | -4.76 (-5.18, -4.34) |  | -4.67 (-5.09, -4.26) |  | -4.43 (-4.85, -4.02) |
| Q4 | -8.32 (-8.93, -7.72) |  | -6.31 (-6.73, -5.88) |  | -6.18 (-6.60, -5.76) |  | -5.86 (-6.28, -5.44) |
| *P* for trend | <0.001 |  | <0.001 |  | <0.001 |  | <0.001 |
| **For VFM** |  |  |  |  |  |  |  |
| **MQI as continuous** | -1.67 (-1.78, -1.57) |  | -1.67 (-1.76, -1.57) |  | -1.62 (-1.71, -1.52) |  | -1.47 (-1.56, -1.37) |
| **MQI as quartile** |  |  |  |  |  |  |  |
| Q1 | 1.00 (ref) |  | 1.00 (ref) |  | 1.00 (ref) |  | 1.00 (ref) |
| Q2 | -1.21 (-1.40, -1.01) |  | -1.28 (-1.45, -1.11) |  | -1.23 (-1.39, -1.07) |  | -1.11 (-1.27, -0.95) |
| Q3 | -1.91 (-2.11, -1.72) |  | -1.96 (-2.13, -1.79) |  | -1.88 (-2.04, -1.71) |  | -1.69 (-1.85, -1.52) |
| Q4 | -2.70 (-2.89, -2.50) |  | -2.69 (-2.87, -2.52) |  | -2.61 (-2.78, -2.44) |  | -2.37 (-2.54, -2.21) |
| *P* for trend | <0.001 |  | <0.001 |  | <0.001 |  | <0.001 |

Abbreviations: CI confidence interval, MQI muscle quality index.

**Supplementary Table S**7. Association of body fat percentage and visceral fat mass with hyperuricemia (n = 5,198).

| **Variables** | **Model 1** |  | **Model 2** |  | **Model 3** |  | **Model 4** |
| --- | --- | --- | --- | --- | --- | --- | --- |
|  | **OR (95%CI)** |  | **OR(95%CI)** |  | **OR (95%CI)** |  | **OR (95%CI)** |
| **BF% as continuous** | 1.02 (1.01, 1.03) |  | 1.11 (1.10, 1.13) |  | 1.03 (1.02, 1.04) |  | 1.03 (1.02, 1.04) |
| **BF% as quartile** |  |  |  |  |  |  |  |
| Q1 | 1.00 (ref) |  | 1.00 (ref) |  | 1.00 (ref) |  | 1.00 (ref) |
| Q2 | 1.56 (1.26, 1.93) |  | 2.05 (1.64, 2.55) |  | 1.69 (1.35, 2.12) |  | 1.58 (1.26, 1.98) |
| Q3 | 1.07 (0.85, 1.34) |  | 3.01 (2.33, 3.90) |  | 1.39 (1.09, 1.76) |  | 1.30 (1.02, 1.66) |
| Q4 | 1.47 (1.19, 1.82) |  | 8.84 (6.38, 12.23) |  | 2.10 (1.66, 2.66) |  | 1.89 (1.49, 2.41) |
| *P* for trend | 0.029 |  | <0.001 |  | <0.001 |  | <0.001 |
| **VFM as continuous** | 1.24 (1.21, 1.27) |  | 1.33 (1.29, 1.37) |  | 1.34 (1.29, 1.38) |  | 1.33 (1.28, 1.38) |
| **VFM as quartile** |  |  |  |  |  |  |  |
| Q1 | 1.00 (ref) |  | 1.00 (ref) |  | 1.00 (ref) |  | 1.00 (ref) |
| Q2 | 1.92 (1.44, 2.56) |  | 2.23 (1.66, 3.00) |  | 2.29 (1.69, 3.09) |  | 2.26 (1.67, 3.06) |
| Q3 | 3.90 (2.99, 5.09) |  | 5.63 (4.23, 7.48) |  | 5.72 (4.25, 7.70) |  | 5.47 (4.06, 7.38) |
| Q4 | 5.79 (4.47, 7.51) |  | 10.23 (7.60, 13.77) |  | 10.95 (8.01, 14.98) |  | 9.91 (7.21, 13.62) |
| *P* for trend | <0.001 |  | <0.001 |  | <0.001 |  | <0.001 |

Abbreviations: OR Odds Ratio, CI confidence interval, MQI muscle quality index, BF% body fat percentage, VFM visceral fat mass.
